# Acquired glucose‐6‐phosphate dehydrogenase deficiency after allogeneic stem‐cell transplantation

**DOI:** 10.1002/jha2.920

**Published:** 2024-06-30

**Authors:** Quentin Vô, Mehdi Khourssaji, Alaa Beshir, Elodie Collinge

**Affiliations:** ^1^ CHU UCL Namur, Hematology department Yvoir Belgium; ^2^ CHU UCL Namur Clinical Biology Yvoir Belgium

**Keywords:** G6PD, red cell enzymopathies, stem cell transplantation

1

A 29‐year‐old Caucasian man was treated for acute myeloid leukemia. He underwent allogeneic stem cell transplantation from an unrelated donor. Six months after the transplant, he relapsed, and salvage chemotherapy was administered, including azacitidine and venetoclax. Vitamin C was added to enhance the azacitidine effect. Rasburicase and allopurinol were given to prevent tumor lysis syndrome. The following day, the patient developed a fever, and an influenza infection was diagnosed and treated with oseltamivir.

On the third day of azacitidine treatment, his blood test showed a hemoglobin level of 6.8 g/dL (range: 13.3–17.6), mean corpuscular volume of 110.6 fL (range: 80.1–99.8), indirect hyperbilirubinemia (indirect bilirubin at 6.35 mg/dL), an undetectable haptoglobin, and a high level of lactate dehydrogenase (1464 IU/L, range: 120–246). These features were suggestive of a hemolytic crisis. A blood smear was performed, showing no schizocytes but revealing hemighost red blood cells (Figure 1), suggesting a glucose‐6‐phosphate dehydrogenase (G6PD) deficiency. Other causes of hemolysis were excluded, including deficiencies and thrombotic microangiopathy.

**FIGURE 1:**
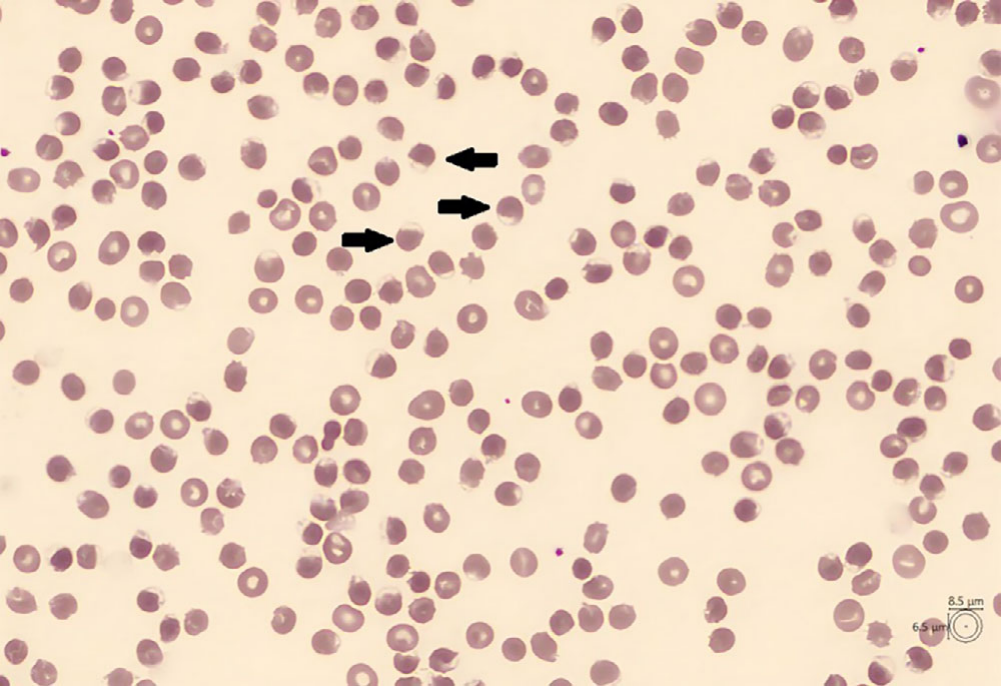
Blood smear exhibiting hemighost cells (Arrows). Hemighost, also known as blister cells, are typically observed in instances of oxidative stress, particularly in cases of glucose‐6‐phosphate dehydrogenase (G6PD) deficiency. Damaged hemoglobin contracts and gathers on one side of the cell, creating an empty space.

The patient had no personal or family history of a hemolytic crisis. The G6PD activity was low, but the patient had received red blood cell transfusions. The graft donor came from the Middle East. After a few days, the G6PD deficiency was confirmed by the donor medical team, but this information was not transmitted before the allogeneic stem cell transplantation.

G6PD deficiency, also known as favism, is an X‐linked hereditary disease commonly found in African, Asian, Mediterranean, and Middle Eastern regions. It is the most common human enzyme defect, affecting more than 500 million people worldwide. Hemolysis mainly occurs after infection or exposure to oxidant drugs. Rasburicase is known to be a strong trigger of hemolysis in G6PD‐deficient patients.

To our knowledge, this represents the first documented instance of a hemolytic crisis arising from acquired G6PD deficiency subsequent to an allogeneic stem cell transplantation. G6PD deficiency may be transmitted either from a symptomatic donor, regardless of gender, or from an asymptomatic heterozygous female donor following lyonization. The case report highlights the difficulty of establishing a diagnosis of a congenital disease, especially in a low‐prevalence country. It underscores the importance of a correct donor evaluation and the necessity of good communication between the patient/donor transplant teams.

## AUTHOR CONTRIBUTIONS

Dr. Vô Quentin: data collection, writing, and editing of the manuscript. Dr. Beshir Alaa: data collection. Dr. Collinge Elodie: critical feedback and manuscript revision. Dr. Khourssaji Mehdi: biological analysis and clinical photography. All authors read and approved the final manuscript.

## CONFLICT OF INTEREST STATEMENT

The authors declare no conflict of interest.

## FUNDING INFORMATION

The authors received no specific funding for this work.

## ETHICS STATEMENT

The authors have confirmed ethical approval statement is not needed for this submission.

## PATIENT CONSENT STATEMENT

The authors have confirmed patient consent statement is not needed for this submission.

## CLINICAL TRIAL REGISTRATION

The authors have confirmed clinical trial registration is not needed for this submission.

## Data Availability

Not Applicable.
